# Primary psoas abscess extending to thigh adductors: case report

**DOI:** 10.1186/1471-2474-11-176

**Published:** 2010-08-06

**Authors:** Zhongjie Zhou, Yueming Song, Qianyun Cai, Jiancheng Zeng

**Affiliations:** 1 Department of Orthopaedics, West China Hospital, Sichuan University, Guoxue road, Chengdu, China; 2 Department of Pediatrics, West China Second University Hospital, Sichuan University, Renmin South Road, Chengdu, China

## Abstract

**Background:**

Psoas abscess is a rare condition consisting of pyomyositis of the psoas. The worldwide incidence was 12 cases per 100,000 per year in 1992, but the current incidence is unknown. Psoas abscess can descend along the psoas sheath and reach the inner upper third of the thigh, but only infrequently does it penetrate the sheath and involve the thigh adductors. Because of insidious clinical presentation, the diagnosis of psoas abscess is a challenge. Delayed diagnosis can result in poor prognosis.

**Case presentation:**

A 45-year-old male with no significant past medical history presented with pain in the left thigh, and limitation of movement at the left hip and knee joint for one month. Ultrasound, CT, and MRI revealed a liquid mass in the left psoas. Percutaneous drainage of this mass yielded 300 ml pus from the psoas. After surgery, the patient reported relief of pain; however, ten days after removal of the drainage tube, the patient complained of persistent pain in his left thigh. CT revealed that the psoas abscess had extended inferiorly, and involved the entire set of adductors of the left thigh. Open surgical drainage was performed at the flank and at the thigh, yielding 350 ml of pus from the thigh. After open drainage and adequate antibiotic therapy, the patient made a good recovery. Follow-up CT confirmed complete resolution of the abscess.

**Conclusions:**

Large psoas abscess can penetrate the psoas sheath, and descend to thigh adductors even after percutaneous drainage. Appropriate treatment includes open surgical drainage along with antibiotic therapy.

## Background

The psoas muscle is a retroperitoneal muscle that originates from the lateral borders of the 12th thoracic to fifth lumbar vertebrae, and ends as a tendon that inserts into the lesser trochanter [1]. Psoas abscess, first described by Mynter in 1881 as "psoitis"[2], is a collection of pus in the psoas compartment, and can be primary or secondary. The clinical presentation is variable and often, non-specific. Classic presentation is characterized by fever, back pain, and anterior thigh or groin pain. The diagnosis is aided by radiologic testing; CT is useful in making a definitive diagnosis, but ultrasound also can be a good choice to detect a large psoas abscess. Treatment involves the use of appropriate antibiotics along with drainage of the abscess. With appropriate treatment, the prognosis is good[3]. Psoas abscess can descend along the psoas sheath and reach the inner upper third of the thigh, but it infrequently penetrates the sheath and involves thigh adductors. The patient reported here had this rare complication, which was treated successfully with open surgical drainage and antibiotic therapy.

## Case presentation

A 45-year-old man presented with pain in the left thigh and limitation of movement at the left hip and knee joint for one month. Ten days prior to presentation, his symptoms had worsened, and he began to have flank pain. He was afebrile. He had no history of diabetes mellitus, neoplasm, or gastrointestinal or genitourinary disease. The pain exacerbated when he lifted his left thigh against the examiner's hand. He reported no tenderness to palpation or percussion of his lumbar vertebrae. Laboratory studies showed an elevated sedimentation rate (ESR) of 55 mm/h, white cell count (WBC) 10.4 × 109/L and C-reactive protein (CRP) 183 mg/L. Stool and urine tests were normal.

**Figure 1:**
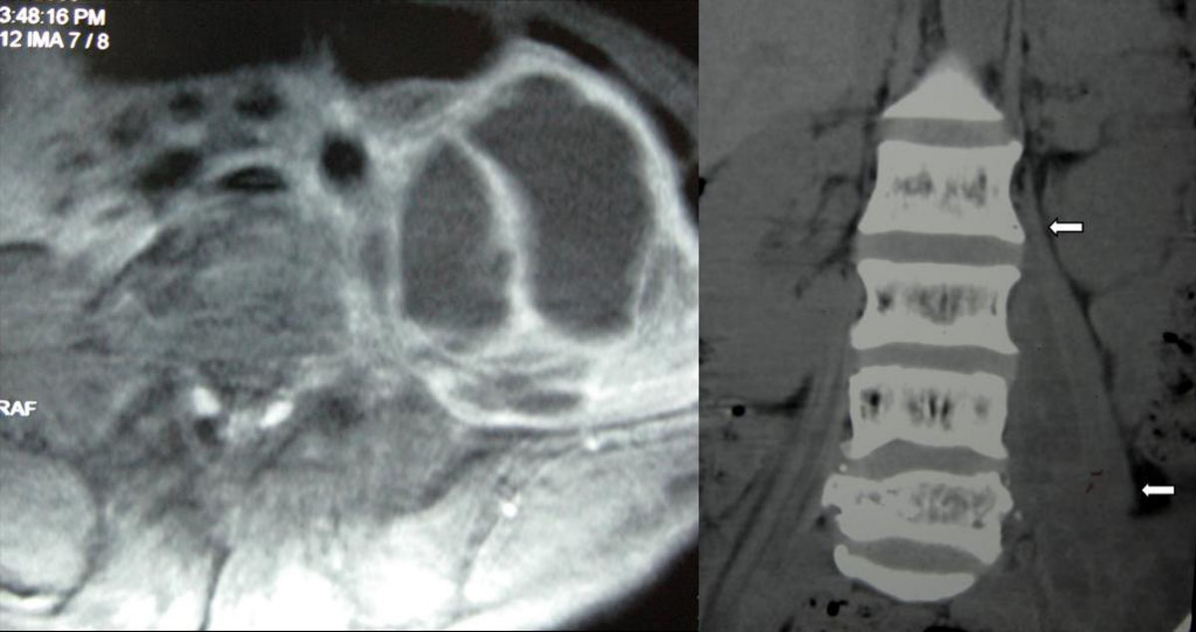
**MRI and CT showing the large lesion in the left psoas**.

One week after he was admitted to the hospital, the patient became febrile to >39°C. Ultrasound revealed a bulky, non-uniform hypoechoic mass anterior to the left hip, which extended intra-abdominally up to the iliac fossa, and to the position of the psoas muscle. CT and MRI of the abdomen and pelvis revealed a large lesion with abnormal signal intensities in the left psoas muscle (Figure 1). MRI and emission computerized tomography (ECT) excluded spondylodiscitis and osteomyelitis. Ultrasound guided percutaneous drainage yielded 300 ml of pus from the psoas muscle; culture of this material detected Staphylococcus aureus. Blood culture was negative.

**Figure 2:**
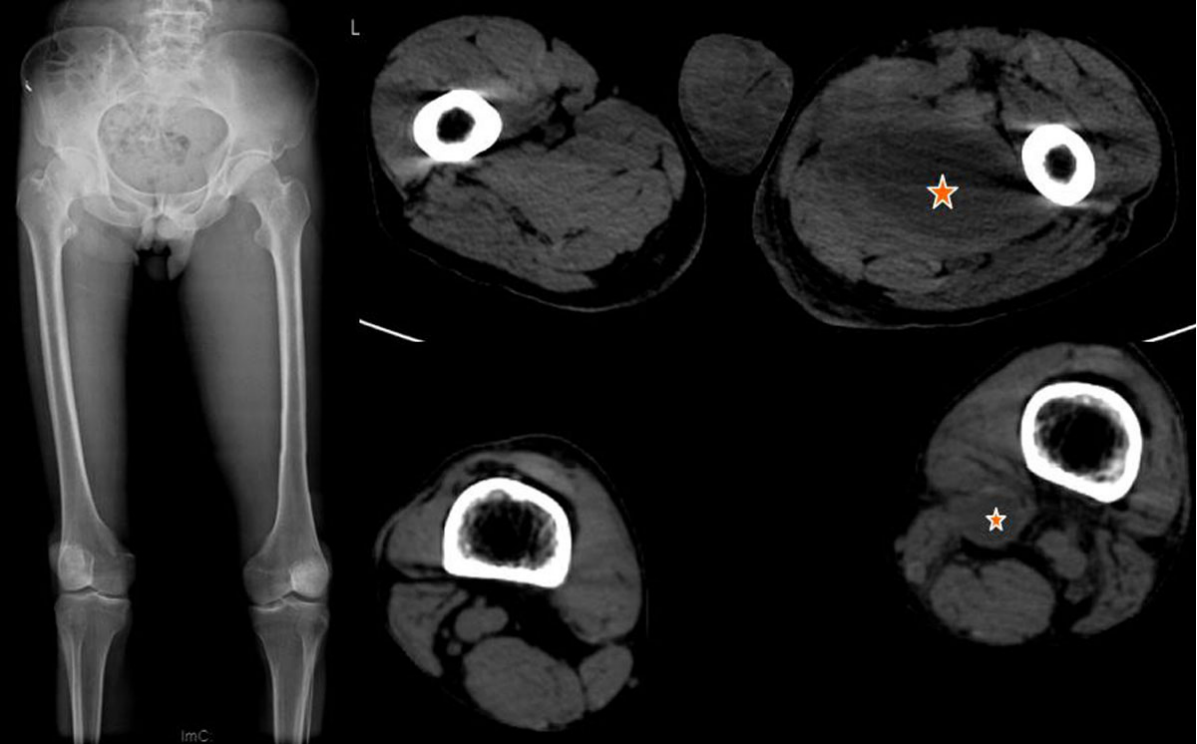
**Plain film and CT of the lower limbs comparing the two sides**. Plain film shows that medial aspect of the left thigh is swelling; CT shows the abscess involves left thigh adductors while the right thigh is normal.

Penicillin (3.2 million units twice daily) and levofloxacin (2 g twice daily) were given. A drain was left in situ after the percutaneous drainage, and was removed 5 days later when the catheter had had no output for 2 consecutive days. After the surgery, the patient reported relief of pain. His temperature returned to normal; however, ten days later, the patient complained of persistent pain in his left thigh. Plain film and CT scan revealed that the abscess had extended to the thigh adductors extending almost to the knee (Figure 2), even while the abscess in the psoas muscle had decreased in size.

Open surgical drainage was performed at the flank and at the thigh; yielding 350 ml of pus from the thigh. Postoperative drain was removed 10 days later when the catheter output was nil for 2 consecutive days. Antibiotics were administered for 3 weeks, and the patient made a good recovery. Follow-up CT scan performed one month after surgery revealed complete resolution of the abscess.

## Discussion

Psoas abscess, a condition that can be treated successfully by drainage and administration of appropriate antibiotics, has been reported more commonly in recent years. Rarely, psoas abscess can extend to the adductors of the lower extremities, as we found (unilaterally) in our patient. Chalaupka reported a 77-year-old woman with concomitant abscesses of the psoas and adductor brevis muscles[4]. In our case, the abscess extended farther, involving the entire set of adductors of the left thigh.

The clinical presentation of iliopsoas abscess is variable and often non-specific[1]. Our patient presented with pain in the left thigh and limitation of movement at the left hip and knee joint, which may be confused with neurological or joint disease. Because the psoas muscle is innervated by L2, L3, and L4 nerve roots, pain can radiate to the hip and thigh. Imaging investigations are helpful in making a definitive diagnosis. Computed tomography is considered to be the gold standard[5].

Psoas abscess may occur as a primary infection of the psoas muscle, or as a secondary abscess from the direct extension of infection from adjacent organs[5]. Our patient did not have any gastrointestinal or genitourinary symptoms. Stool and urine tests were normal. MRI and ECT excluded spondylodiscitis and osteomyelitis. We believe that the patient had a primary psoas abscess.

Having reviewed 367 cases of psoas abscess, Ricci et al. observed that *Staphylococcus aureus *is the causative organism in over 88% of patients with primary psoas abscess, and that secondary psoas abscess is usually caused by enteric bacteria[6]. These conclusions are generally accepted. In our case, *Staphylococcus aureus *was the causative organism. Lopez et al. analyzed 124 cases collected from 1990 to 2004 in Spain and found that *S. aureus *is the most common organism in patients with primary abscesses (42.9%) and with abscesses of skeletal origin (35.2%), whereas *E. coli *is the leading organism in those with abscesses of urinary (61.5%) and gastrointestinal (42.1%) tracts. *Mycobacterium tuberculosis *is an important pathogen of psoas abscess secondary to vertebral osteomyelitis[3]. Knowledge of the microbiology and the site of origin is helpful in choosing antibiotics before the results of culture and sensitivity testing become available.

Prassopoulos et al. reported a newborn with primary psoas abscess, who was treated successfully with antibiotic therapy alone[7]; however, for most patients, treatment should include the use of appropriate antibiotics along with drainage of the abscess[3-6]. The choice of drainage may be surgical, or radiologically guided percutaneous drainage. For secondary psoas abscess, surgical drainage can be combined with treatment of the primary (causative) disease. In the case of primary psoas abscess, percutaneous drainage is indicated for a small, and single abscess if technically possible; however, for large, extensive, or multiple abscesses, percutaneous drainage may result in recurrence or simply be insufficient. In this situation, open surgical drainage is more appropriate[8].

In our case, the abscess was large and extended to the thigh, and it descended to the adductors after percutaneous drainage. It is possible that early open drainage could avoid this complication. Eventually, open surgery was performed for a thorough drainage, and the patient finally made a full recovery.

## Conclusions

The abscess in our patient was large, and extended to the thigh adductors, thus demonstrating an additional route of extension of psoas abscess. Ultrasound, CT, and MRI helped to make a definitive diagnosis. The patient was treated successfully with open surgical drainage and antibiotic therapy.

## Consent

Written informed consent was obtained from the patient for publication of this case report and any accompanying images. A copy of the written consent is available for review by the Editor-in-Chief of this journal.

## Competing interests

The authors declare that they have no competing interests.

## Authors' contributions

ZZ, YS, JZ participated in diagnosing and treating the patient, acquisition of data, literature review, and drafting the manuscript. QC was involved in acquisition of data and literature review, as well as drafting and revising the manuscript. All authors read and approved the final manuscript.

## Pre-publication history

The pre-publication history for this paper can be accessed here:

http://www.biomedcentral.com/1471-2474/11/176/prepub
